# Automated sleep stage classification based on tracheal body sound and actigraphy 

**DOI:** 10.3205/000268

**Published:** 2019-02-22

**Authors:** Christoph Kalkbrenner, Rainer Brucher, Tibor Kesztyüs, Manuel Eichenlaub, Wolfgang Rottbauer, Dominik Scharnbeck

**Affiliations:** 1 Faculty of Medical Engineering, University of Applied Science Ulm, Germany; 2 Institute of Medical Systems Biology, University Ulm, Germany; 3 School of Engineering, University of Warwick, Coventry, United Kingdom; 4 Department of Internal Medicine II, University Hospital Ulm, Germany

**Keywords:** sleep staging, monitoring, respiratory sounds, movement analysis

## Abstract

The current gold standard for assessment of most sleep disorders is the in-laboratory polysomnography (PSG). This approach produces high costs and inconveniences for the patients. An accessible and simple preliminary screening method to diagnose the most common sleep disorders and to decide whether a PSG is necessary or not is therefore desirable. A minimalistic type-4 monitoring system which utilized tracheal body sound and actigraphy to accurately diagnose the obstructive sleep apnea syndrome was previously developed. To further improve the diagnostic ability of said system, this study aims to examine if it is possible to perform automated sleep staging utilizing body sound to extract cardiorespiratory features and actigraphy to extract movement features.

A linear discriminant classifier based on those features was used for automated sleep staging using the type-4 sleep monitor. For validation 53 subjects underwent a full-night screening at Ulm University Hospital using the developed sleep monitor in addition to polysomnography. To assess sleep stages from PSG, a trained technician manually evaluated EEG, EOG, and EMG recordings.

The classifier reached 86.9% accuracy and a Kappa of 0.69 for sleep/wake classification, 76.3% accuracy and a Kappa of 0.42 for Wake/REM/NREM classification, and 56.5% accuracy and a Kappa of 0.36 for Wake/REM/light sleep/deep sleep classification. For the calculation of sleep efficiency (SE), a coefficient of determination r^2^ of 0.78 is reached. Additionally, subjects were classified into groups of SEs (SE≥40%, SE≥60% and SE≥80%). A Cohen’s Kappa >0.61 was reached for all groups, which is considered as substantial agreement.

The presented method provides satisfactory performance in sleep/wake and wake/REM/NREM sleep staging while maintaining a simple setup and offering high comfort. This minimalistic approach may address the need for a simple yet reliable preliminary sleep screening in an ambulatory setting.

## Zusammenfassung

Der aktuelle Goldstandard für die Beurteilung der meisten Schlafstörungen ist die Polysomnographie (PSG). Diese Methode der Diagnose ist mit hohen Kosten und Unannehmlichkeiten für die Patienten verbunden. Eine einfache Methode der Diagnose der häufigsten Schlafstörungen ist daher wünschenswert. Hierzu wurde ein minimalistischer Typ-4-Schlafmonitor, welcher Körperschall und Aktigraphie zur Diagnose des obstruktiven Schlafapnoe-Syndroms einsetzt, entwickelt. Um die Diagnosefähigkeit dieses Systems zu erweitern, soll in dieser Studie untersucht werden, ob der Schlafmonitor automatisiert Schlafstadien klassifizieren kann. Hierbei wird Körperschall verwendet, um kardiorespiratorische Merkmale zu extrahieren, und Aktigraphie, um Bewegungsmerkmale zu extrahieren.

Ein auf diesen Merkmalen basierender linearer Diskriminanzklassifizierer wurde für die automatisierte Klassifizierung von Schlafstadien mit dem vorgestellten Typ-4-Schlafmonitor verwendet. Zur Validierung wurden 53 Probanden am Universitätsklinikum Ulm zusätzlich zur PSG einem nächtlichen Screening mit dem entwickelten Schlafmonitor unterzogen. Zur Beurteilung der Schlafstadien der PSG hat ein geschulter Techniker EEG-, EOG- und EMG-Aufnahmen manuell ausgewertet.

Der Klassifikator erreichte eine Genauigkeit von 86,9% und ein Kappa von 0,69 für Schlaf/Wach-Klassifizierung, 76,3% Genauigkeit und ein Kappa von 0,42 für Wach/REM/NREM-Klassifizierung, und 56,5% Genauigkeit und ein Kappa von 0,36 für Wach/REM/Leichtschlaf/Tiefschlaf-Klassifizierung. Für die Berechnung der Schlafeffizienz (SE) wird ein Bestimmtheitsmaß r^2^ von 0,78 erreicht. Zusätzlich wurden die Probanden in Gruppen von SEs eingeteilt (SE≥40%, SE≥60% und SE≥80%). Ein Cohen’s Kappa >0,61 wurde für alle Gruppen erreicht, was als substantielle Übereinstimmung angesehen wird.

Die vorgestellte Methode bietet eine zufriedenstellende Leistung in der Schlaf/Wach- und Wach/REM/NREM-Schlaf-Klassifizierung bei einfachem Aufbau und hohem Patientenkomfort. Dieser minimalistische Ansatz kann den Bedarf an einem einfachen aber zuverlässigen Vorab-Schlaf-Screening im ambulanten Bereich abdecken.

## Introduction

The number of individuals suffering from sleep disorders worldwide continually increases at a drastic rate. However, public awareness of the importance of sleep quality and the implications of sleep disorders is low [1]. A good example for this lack of awareness is obstructive sleep apnea (OSA), the most common cardio-respiratory sleep disorder. Here, 10% of 30- to 49-year-old men, 17% of 50- to 70-year-old men, 3% of 30- to 49-year-old women, and 9% of 50- to 70-year-old women suffer from moderate to severe OSA [2]. OSA can lead to cardiovascular diseases and extensive daytime sleepiness. The resulting cognitive impairment often comes with personal and societal consequences, such as driving and workplace accidents [3]. The gold reference for the diagnosis of sleep disorders is in-laboratory polysomnography (PSG). Its labor intensive, expensive, and time-consuming nature, paired with the increasing prevalence of sleep disorders, has led to a strong demand for appropriate hospital facilities. Therefore, sleep centers worldwide are typically operating at full capacity and waiting times are long, causing economic losses due to prolonged invalidity. Patients are often reluctant to carry out a PSG since an overnight stay in an unfamiliar sleep laboratory is required. Furthermore, during the first night the extensive recording often worsens their already bad sleep. A possible solution for these issues would be an accessible and simple preliminary screening method for the most common sleep disorders. Based on the results of this initial test, further diagnostic measures like the PSG could be considered.

Several less extensive sleep diagnostic methods have been developed. The most simple methods use 1–2 recording channels and are referred to as type-4 sleep studies. They benefit from a low price, simple setup, and can often be used in a home setting without medical assistance. In the context of OSA diagnosis, the developed systems either use nasal airflow and/or S_p_O_2_ [4], [5]. However, these measurement channels induce several problems and limitations. Mouth-breathing or misplacement frequently lead to signal loss. Additionally, those systems cannot perform any sleep staging, and systematic reviews reported poor diagnostic performance for OSA [6], [7].

Sleep stages are of great interest for sleep screening since they are used to evaluate total sleep time, measure the overall level of sleep quality, and detect sleep disruptions. Sleep stages can also be used to diagnose other sleep disorders such as insomnia and circadian rhythm disorders [8]. Human sleep can be classified into the stages wake (W), rapid-eye movement (REM), and three non-REM stages (N1, N2, N3) [9]. N1 and N2 are often grouped into so-called “light sleep”, and N3 is often referred to as “deep sleep” [10]. The conventional method for sleep staging is the manual evaluation of the electroencephalogram (EEG) recording carried out during PSG. The EEG comes with several technical challenges and is mostly not fit for use at home or without medical attendance. For ambulatory sleep staging, several automated methods have been developed. These mainly focus on evaluating the variation in heart rate and breathing rate as well as movements. This so-called cardiorespiratory sleep stage classification has extensively been studied in recent years [11], [12], [13] and provides promising results. Here, cardiac features are extracted using electrocardiography (ECG), and respiratory features are extracted using respiratory inductance plethysmography (RIP). Furthermore, studies solely relying on respiratory features to assess sleep stages also showed good correlation (>70%) with sleep stage classification [14], [15], [16]. However, these methods also come with a complex setup and cannot be used without assistance or in a home setting. Thus, a method which preserves the simplicity of a type-4 sleep study while performing reliable OSA diagnosis and sleep staging for a preliminary screening is desirable.

A novel type-4 monitoring system has previously been developed by Kalkbrenner et al. [17], [18]. This monitor utilizes tracheal body sound and actigraphy to screen for OSA [19], [20]. This allows simple setup and high comfort, minimizing the effect on sleep quality while outperforming similar existing ambulatory diagnostic. The assessment of sleep stages could improve the diagnostic ability for a preliminary screening even further, but was not part of previous research. Therefore, this study aims to examine if it is possible to use the presented type-4 monitor to perform automated sleep staging utilizing body sound to extract cardiorespiratory features, and utilizing an inertial measurement unit (IMU) to extract movement features. This minimalistic approach may address the need for simple yet reliable preliminary screening including sleep staging and OSA diagnosis in an ambulatory setting.

## Method

### Subjects

60 adult subjects were included in the present study. All subjects were referred to the sleep center at Ulm University Hospital with suspicion of OSA. During their overnight stay, all subjects underwent full-night diagnostic PSG screening. Simultaneously a recording using the new monitoring system was carried out. Recordings only include so-called diagnostic nights without the presence of any therapeutic measures. Data recorded here was also used in another study to validate the ability of the new monitoring device to screen for OSA [19]. The study was approved by the ethics committee of Ulm University, and all subjects gave written informed consent. 

In total, seven recordings were excluded due to faulty body sound (n=1) and faulty ECG (n=6) recordings. These faulty recordings included subjects suffering from central sleep apnea or mixed forms, and subjects suffering from Cheyne-Stokes respiration. There were no data sets with faulty submental channel recordings. The remaining 53 data sets were considered for sleep staging. Since all subjects were recruited with a suspicion of OSA, 14 patients suffered from mild, 12 from moderate, and 16 from severe OSA. The remaining 11 patients were not diagnosed with OSA. No other sleep disorders were diagnosed in the subjects considered. Detailed anthropometric information of the subjects is shown in Table 1 (Tab. 1).

### Data acquisition

Trained medical staff set up the PSG and the new monitoring system. Recordings were monitored during the night. The recording for the diagnosis started between 9 pm and 11 pm and ended between 5 am and 7 am. PSG was carried out using the SOMNOlab PSG system (Co. Weinmann Geräte für Medizin GmbH + Co. KG, Kronsaalsweg 40, 22525 Hamburg, Germany). EEG included channels C3-A2 and C4-A1 with a sampling rate of 256 Hz. Furthermore, submental EMG, unilateral anterior tibial EMG, and bilateral EOG were included and sampled with 256 Hz. The PSG system also included video recording during the night. To assess sleep stages, a trained technician manually evaluated EEG, EOG, and EMG recordings according to the AASM criteria [21]. Each 30-second epoch is assigned to one sleep stage (WAKE, REM, N1, N2, or N3). The oronasal airflow was recorded by using a thermistor and was sampled with 32 Hz. Additionally, thoracic and abdominal respiratory movements were measured using respiratory inductance plethysmography, sampled with 32 Hz. Oxygen saturation was recorded by using finger pulse oximetry, sampled with 16 Hz.

**Figure 1:**
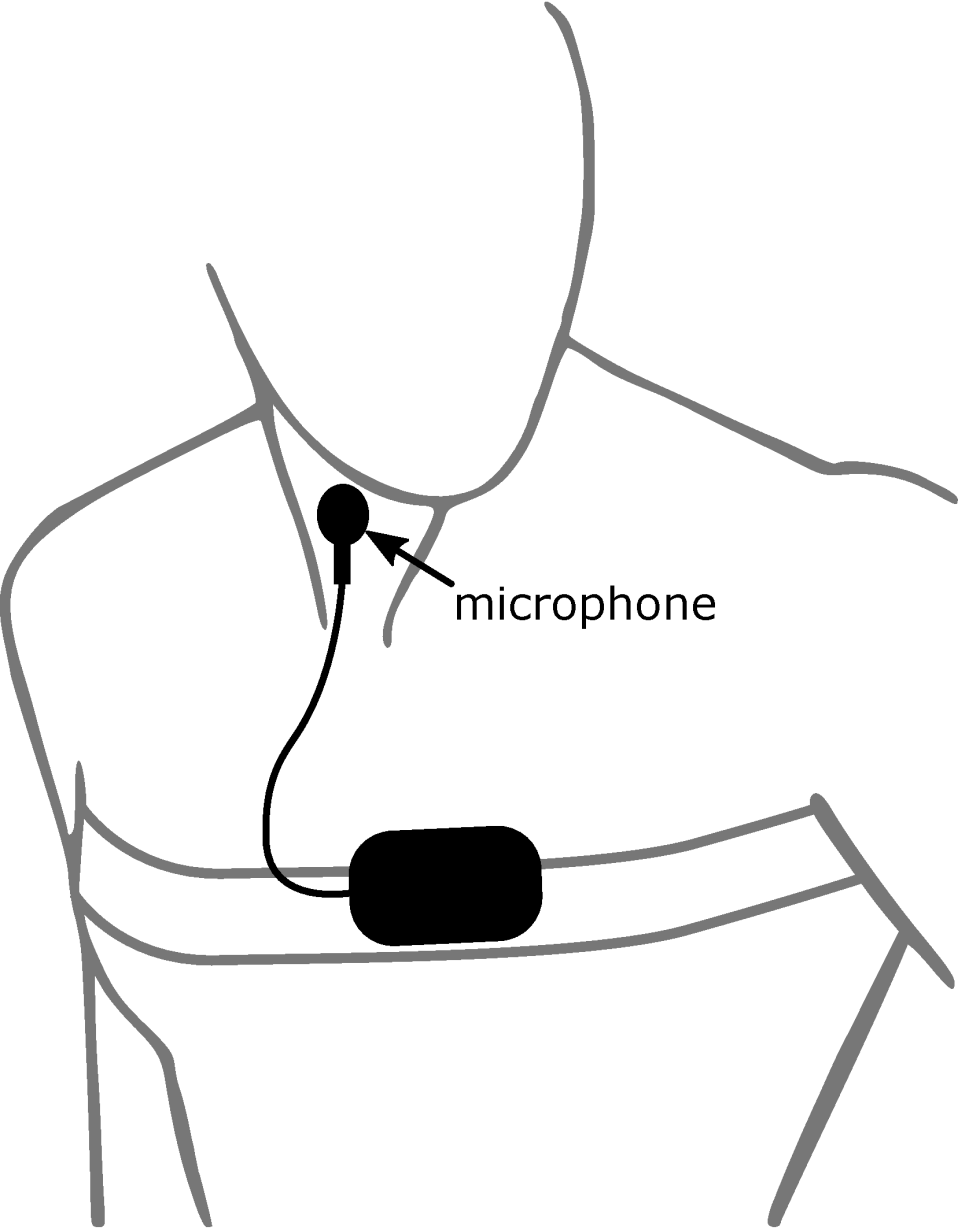
Abstract illustration of the setup of the new sleep monitor system. The microphone is attached on the subject’s neck in close vicinity to the trachea. The remaining hardware is attached to the existing thoracic belt of the respiratory inductance plethysmograph of the polysomnography.

The new monitoring system has previously been described in [17], [18]. This previous research presents the developed monitoring system as a new, reliable, and simplified ambulant sleep monitor, only utilizing tracheal body sound and movement data to automatically diagnose OSA. The main criteria to indicate the severity of OSA is the apnea-hypopnea index (AHI), which the proposed sleep monitor estimates precisely to reliably diagnose sleep apnea and its severity. Figure 1 (Fig. 1) shows an abstract illustration of the setup of this monitor. A commercially available body sound microphone was used to record body sound. It was attached to the subject’s neck and sampled with 5 kHz. The microphone was designed for the long-term monitoring of lung sounds to diagnose breathing disorders like asthma and is part of a system called LEOSound (Co. Heinen+Löwenstein GmbH & Co. KG Arzbacher Straße 80, 56130 Bad Ems, Germany). In addition, an inertial measurement unit (IMU) was implemented as actigraph to record the movements of the subject. The IMU measures acceleration and gravitational forces using a combination of accelerometers and gyroscopes sampled with 250 Hz. The IMU was attached to the existing thoracic belt of the respiratory inductance plethysmograph of the PSG with a defined orientation. This defined orientation is necessary to evaluate sleep position. For subsequent data analysis, all data were transmitted wirelessly to a laptop.

### Feature extraction

A vast amount of signals and their characteristics can be used to classify sleep stages [22]. Using the new monitoring system, breathing cycles, heart beats and movements can be extracted. Various basic research revealed that the dynamics of heart rate [23], [24], [25], [26] and the dynamics of respiration [27], [28] relate to sleep stages. Previous studies presented a vast collection of appropriate cardiorespiratory features for sleep staging. Methods presented by Khalighi et al. were used to choose the optimal features for the proposed sleep staging [29]. Additionally, it is suggested that subject movements may also relate to sleep stages and are therefore included into the feature selection. Each feature and their correspondent source is listed in Table 2 (Tab. 2). The methods to extract the cardiorespiratory features from the tracheal audio signal are described in the following.

### Respiratory

**Figure 2:**
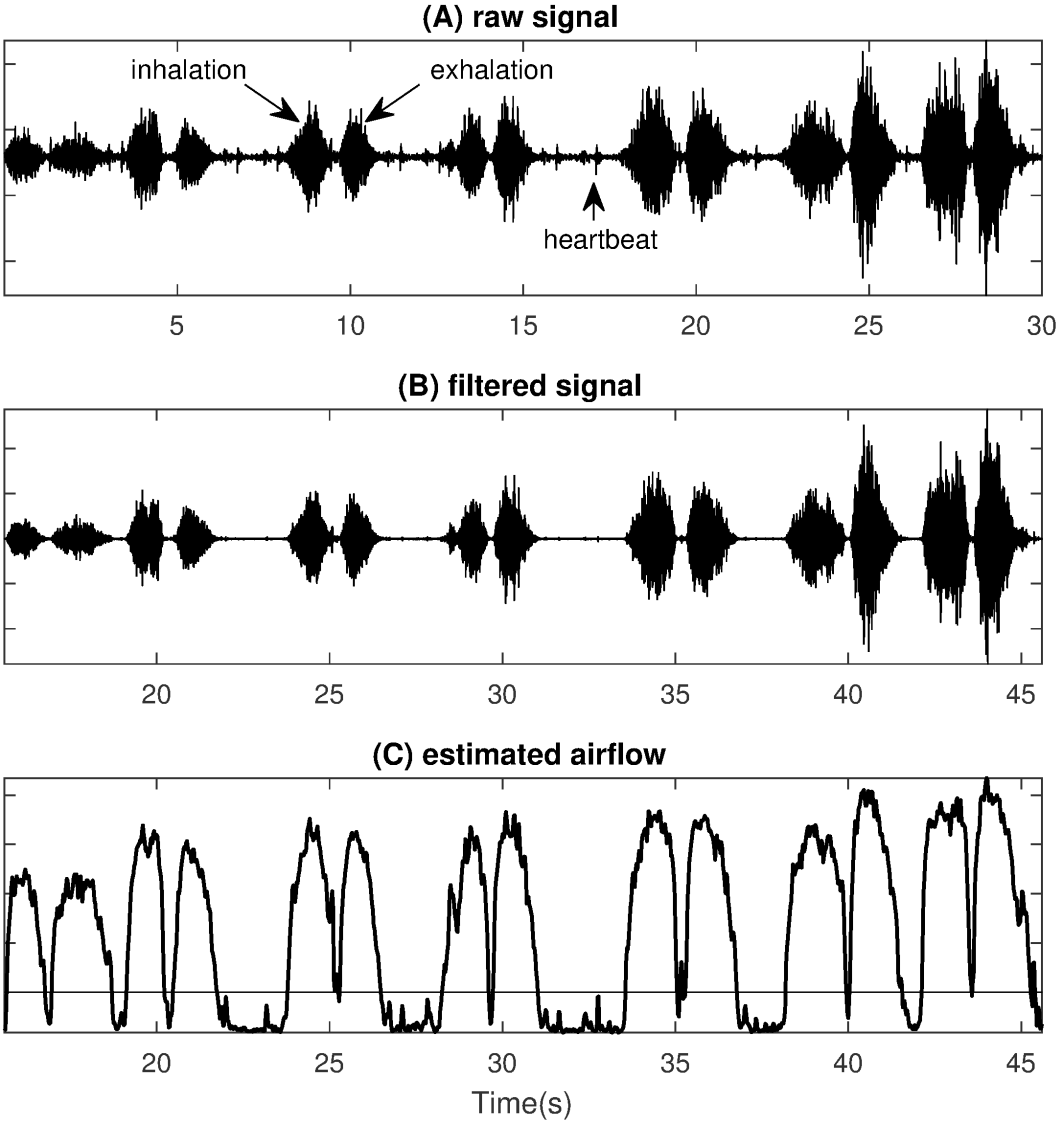
Illustration of the signal processing steps of the tracheal body sound for respiratory feature extraction; (A) raw audio signal; (B) audio signal after FIR-filtering and spectral subtraction; (C) estimation of airflow, values below the horizontal line are considered no breathing.

The tracheal body sound is utilized to extract the respiratory features. Figure 2 (Fig. 2) illustrates the key steps of the developed method. The initial raw audio signal consists of breathing, heart sounds, background noise and movement artefacts. To obtain a pure breathing sound signal, a FIR bandpass filter with boundaries between 200 and 2000 Hz is used. To reduce background noise a noise template is subtracted from the signal in the frequency domain using spectral subtraction [30]. Finally, the signal is divided into short-term windows and the envelope curve *E* is calculated by 


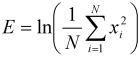


where N is the number of samples in the window and x_i_ the i-th sample.

This curve can now easily be used to detect breathing cycles and estimate airflow. A broader discussion of the challenges of relating the amount of airflow to tracheal breathing sounds can be found in [20], [31], [32]. With this information, the respiratory features presented in Table 2 (Tab. 2) can be calculated. Here, most features relate to the variation of the time interval between successive breaths (BB interval).

### Cardiac

**Figure 3:**
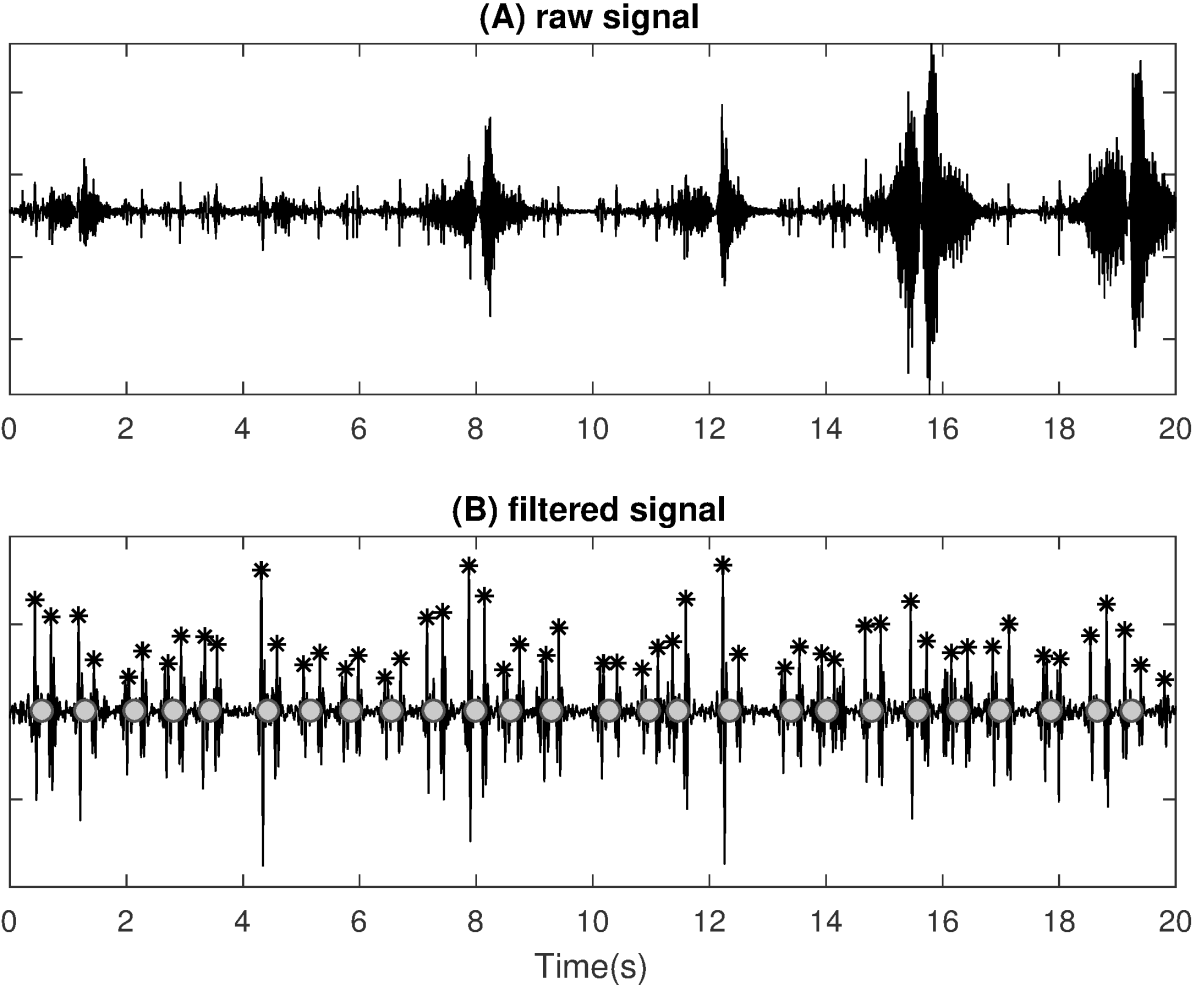
Illustration of the signal processing steps using the tracheal body sound for cardiac feature extraction; (A) raw audio signal, (B) audio signal after filtering in frequency domain; stars mark detected peaks, circles mark detected heart beat consisting of two peaks.

The tracheal body sound signal is also utilized to detect heart beats in order to calculate the appropriate cardiac features. To suppress breathing and most of the artefacts from the initial raw audio signal, a bandpass filter with the boundaries between 5 and 30 Hz is applied. An exemplary raw signal is shown in Figure 3A (Fig. 3) with the correspondent filtered signal shown in Figure 3B (Fig. 3). The filtered signal mostly consists of pairs of distinct peaks generated by physiological heart beats. By searching the minimal distance from each peak to its adjacent peaks, we can group two peaks into one correspondent heartbeat. The presented method enables heart beats to be detected even during snoring. Nevertheless, bad microphone coupling, movements or similar artefacts can cause the heart beat detection to fail. During those periods heart beats are interpolated based on preceding values.

Finally, all cardiac features presented in Table 2 (Tab. 2) are calculated. The extraction of cardiac features mostly focuses on heart rate variability (HRV) analysis, based on evaluation of successive heart beat intervals (NN interval). 

### Movements

The IMU is utilized to extract features considering sleeping position and movements. Methods published by Madgwick et al. [33] are used to process the data of accelerometers and gyroscopes in order to track the orientation of the IMU. Since the IMU is placed at the thoracic belt, the sensor orientation yields information about the movements and the position of the subject. Details of the utilized processing techniques were previously described by Kalkbrenner et al. [18].

Using this information, it is possible to determine the most prominent sleeping position of the subject during one epoch. Additionally, the changes of sleeping position per epoch are counted. To assess the general movement activity of the subject the mean acceleration and angular velocity over all degrees of freedom are calculated.

### Classification

A linear discriminant (LD) classifier is used for automated sleep staging. LD has been extensively and successfully used for sleep staging based on cardiorespiratory signals [11], [12], [15], [16], [34], [35]. Like conventional sleep staging, the classifier assigns every 30-second epoch to one sleep stage. For each epoch, the previously described 30 features can be calculated.

Three different classification systems are defined for sleep staging. The 2-stage system only consists of the stages wake (W) and sleep (combining REM and N1–4). The 3-stage system consists of the stages wake (W), REM (REM) and NREM (N1–3). The 4-stage system consists of the stages wake (W), REM (REM), light sleep (N1, N2), deep sleep (N3). The recorded PSG data was adjusted to the appropriate system accordingly before training and validating the classifier.

### Validation

Since the recorded data is limited, a leave on-out cross validation was carried out to validate the LD classifier instead of simply splitting our data into test and training sets. 53 data sets were created each consisting of 52 (of 53) subjects to train the classifier leaving the remaining subject for testing. For every data set, the subject for testing was changed until every subject has been used for testing once. For final evaluation, the classification accuracy for each sleep stage was computed by averaging over the results from all data sets. The classification accuracy is the percentage of the respective sleep stage correctly classified. Additionally the un-weighted Cohen kappa coefficient [36] was calculated. This coefficient is more applicable to evaluate classifiers for unevenly distributed data like sleep stages. The validation was carried out for the 2-stage, 3-stage and 4-stage system.

One of the essential parts of sleep monitoring, especially in an ambulatory setting, is the measurement of sleep efficiency (SE). The SE is the ratio of sleep time to total time in bed. In this study, the SE is calculated using the results of the 2-stage system classifier (SE_est_) and compared to PSG SE (SE_PSG_) using correlation analysis. Additionally, the wake after onset time (WASO), total sleep time (TST) and total wake time (TWT) are also calculated and compared to the gold standard. 

For expanded use without medical supervision at home, an easy to use indicator for scoring SE is desirable. Therefore, thresholds of SE 0%–39%, SE≥40%–59%, SE≥60%–79% and SE≥80% were defined and subjects were classified into the according groups based on the calculated SE of the 2-stage system classifier. The sensitivity, specificity, positive predictive value, negative predictive value, and the un-weighted Cohen kappa coefficient [36] of these classifications were calculated. Receiver operating characteristic (ROC) curves and the according areas under the curves (AUCs) were calculated to evaluate the performance against the PSG results.

## Results

**Figure 4:**
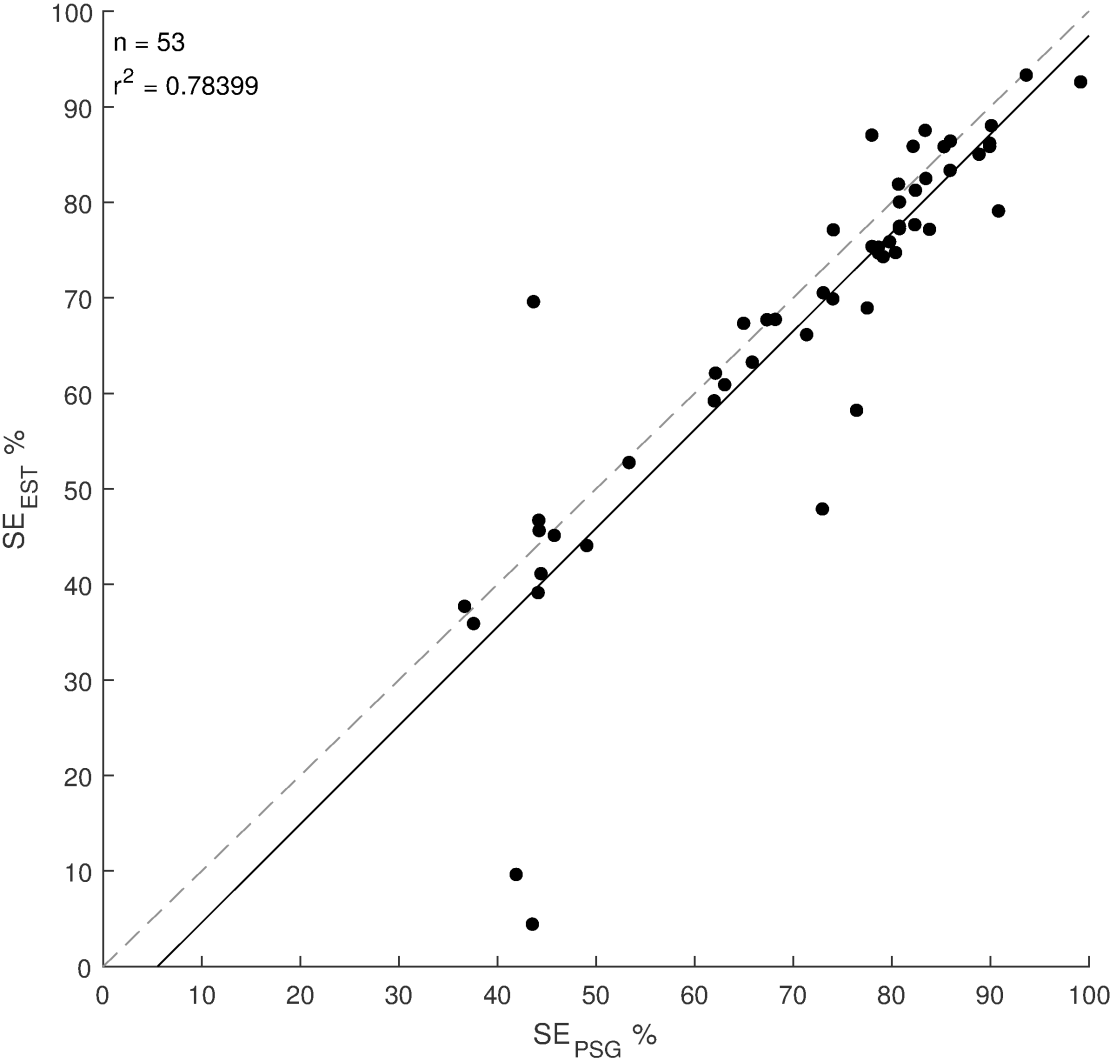
Relationship between sleep efficiency of the new sleep monitor (SE_est_) and the sleep efficiency of the polysomnography (SE_PSG_); r2: coefficient of determination; n: number of data points

**Figure 5:**
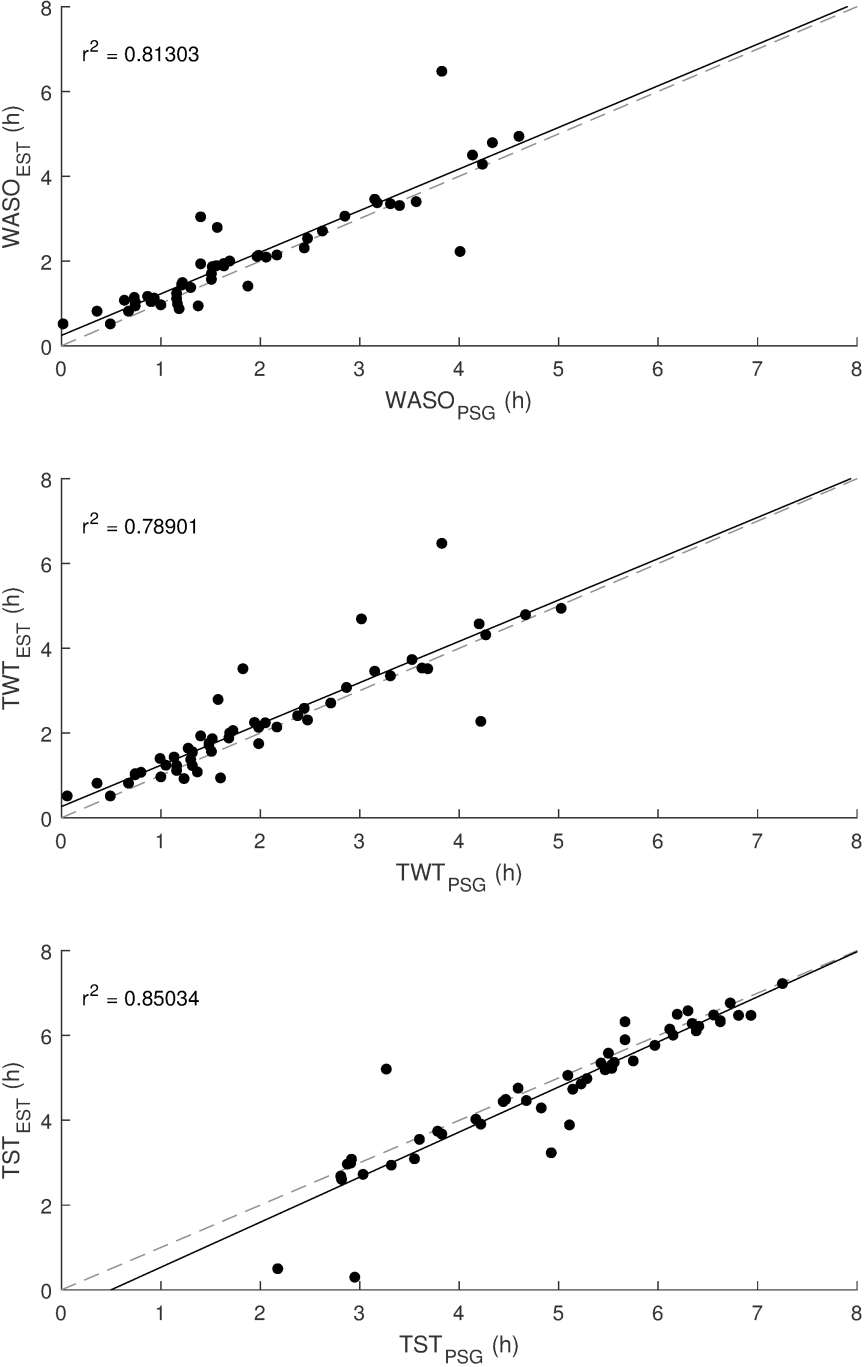
Relationship between wake after onset (WASO), total wake time (TWT) and total sleep time (TST) of the new sleep monitor and the polysomnography including all 53 subjects; r^2^: coefficient of determination

Table 3 (Tab. 3) shows detailed results of the classifier performance of all three staging systems. For each subject the 2-stage classifier result was used to calculate SE_est_. These results were compared to the SE_PSG_. Figure 4 (Fig. 4) shows the corresponding correlation plot. The coefficient of determination r^2^ is 0.78. A standard t-test of the paired differences revealed p=0.008, 95% CI=[0.94 5.98], SD=9.14. Furthermore, Figure 5 (Fig. 5) shows the correlation of the parameters WASO, TWT and TST.

**Figure 6:**
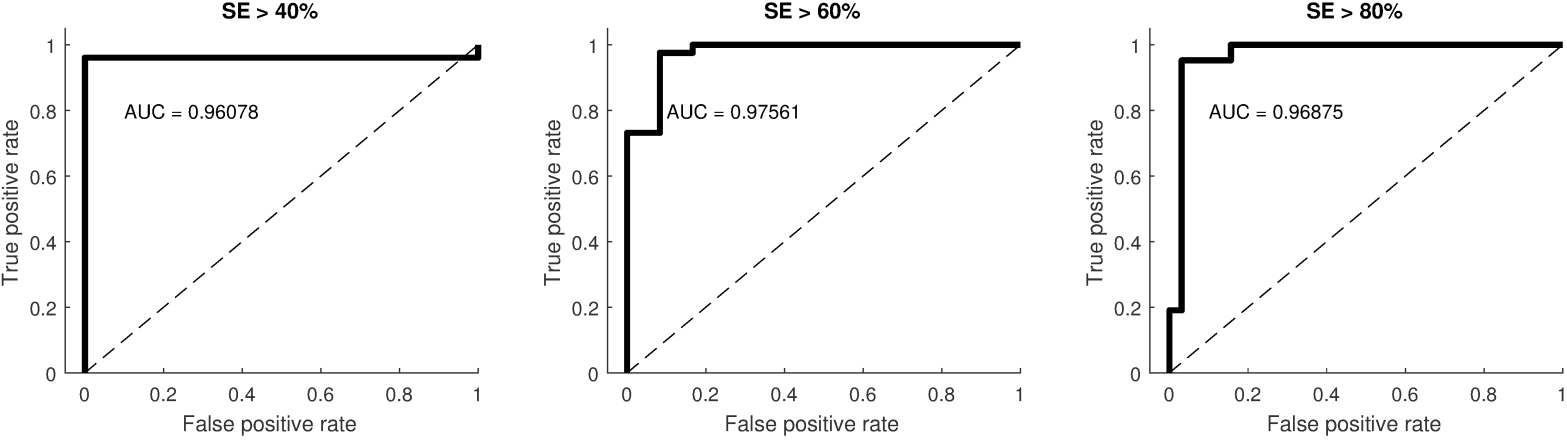
Receiver operating characteristic (ROC) of subject classification into groups of sleep efficiency (SE>40%, >60%, >80%); AUC: area under curve

A detailed performance evaluation of the subject classification into groups of SE≥40%, SE≥60%, and SE≥80% is shown in Table 4 (Tab. 4). A Cohen’s Kappa >0.61 was reached for all groups, which is considered as substantial agreement [19]. The according ROC curves were created and are shown in Figure 6 (Fig. 6).

## Discussion

The application of a new type-4 sleep monitor based on tracheal body sound and movement data for automated sleep staging was demonstrated and its performance was validated by comparison against standard PSG sleep staging. The system is designed to allow a simple setup and high comfort, minimizing its impact on sleep quality. It facilitates ambulatory use with no need of medical supervision for a preliminary sleep screening including sleep staging and OSA diagnosis. Three types of sleep stage classifiers were implemented and validated.

It is important to note that current research suggests a general performance limitation of sleep staging based on cardiorespiratory signals caused by subject variability [37]. Additionally, the sleep staging of the PSG was done manually and is therefore open to the subjectivity of the scoring technician. However, since the evaluation of the sleep stages was carried out within the daily business of the sleep laboratory, we were not able to evaluate the intrarater reliability. Future studies must include this evaluation in order to eliminate the uncertainty of the scoring technician. As shown in Table 4 (Tab. 4), the new sleep monitor provided a substantial agreement with the PSG results in classifying subjects into groups of SE. Table 5 (Tab. 5) shows the results of similar approaches of sleep staging using cardiorespiratory features. It is important to note that those previously proposed methods utilize well established approaches to record cardiorespiratory signals (e.g. ECG, inductance plethysmography). This comparison reveals that the 2- and 3-stage system achieved acceptable results. The 4-stage system gets clearly outperformed by the best results found in literature. Nevertheless, the novel method presented in this paper only uses a type-4 monitor using tracheal body sound recorded with a single lead and movement data to extract all features presented in Table 2 (Tab. 2). Furthermore, the most essential parameters for sleep staging like SE can be calculated using the 2-stage system. It is suggested that the 2- or 3-stage system suffice for a preliminary screening.

Some researchers also present methods for sleep staging utilizing unobtrusive and comfortable methods. Samy et al. present a high-resolution pressure-sensitive bed sheet to extract sleep-related biophysical and geometric features for sleep staging [38]. An overall accuracy of 71.1% was reached for a sleep 3-stage system while including seven subjects in their study. Sensor foils placed into the bed mattress are used by Kortelainen et al. [39] to extract relevant features and parameters for sleep staging. An overall accuracy of 79% and a Kappa of 0.44 was reached for a sleep 3-stage system while including 18 subjects in their study. These results are similar to those presented in this paper. While offering non-contact and unobtrusive sleep staging, the main drawback of those methods are the excessive noise problems during body movements preventing reliable sleep staging. Additionally, the significance of these studies may be limited by the small number of participants.

The present study holds several advantages and limitations. The setup of the PSG and developed monitor was performed by previously trained medical staff. EEG derivatives do not comply with the current AASM standard. However, it can be assumed that the use of several EEG derivatives only leads to minor changes in the distribution of the derived sleep stages and no significant differences in scoring reliability [40]. The PSG results are covering the entire spectrum from healthy subjects to subjects suffering from severe OSA. Additionally, the sex, age, and BMI distribution cover a wide range of different individuals. All subjects included in the present study were recruited with a suspicion of OSA. The sleep of subjects suffering from OSA is disrupted by arousals caused by breathing pauses. Therefore, at least 42 patients included in the present study do not represent a healthy sleep. This may suggest that the presented results are not applicable to the general population. However, 11 subjects included in the presented study did not suffer from OSA. A relationship between OSA and sleep staging performance could not be observed. This finding might indicate that the presented method performs as well for healthy subjects as for subjects suffering from OSA. Redmond et al. [11], [12] also suggest that OSA is no limitation for using cardiorespiratory signals for sleep staging. However, further studies are required to fully validate this statement.

To further improve automated sleep staging, subject-specific classifiers or subject-specific feature normalization are utilized in various research [12], [15]. In general, a subject-independent classifier can be set up without calibration or any adjustment, again facilitating the use in the homecare area without medical supervision. Nevertheless, subject-specific feature normalization or classifier training can be useful in multi-night studies. Further research should be undertaken to investigate the potential of the classifier in subject-specific classification. 

Cardiorespiratory features used for classification in this work are solely based on existing research. Tracheal body sound might offer additional features related to sleep phases currently not utilized. It can be assumed that it is possible to extract snoring, wheezing, and similar breath related sounds. Furthermore, since the new sleep monitor is placed at the thoracic belt of the subject, the movements of the chest due to breathing are also reflected within the IMU data. Therefore, the IMU could be utilized to calculate features relating to thoracic or abdominal respiratory movements.

In comparison to PSG or ECG, there is no need to apply a vast number of additional sensors or electrodes during the night to conduct simple sleep staging and evaluation of SE. Additionally, it is suggested that using less sensors leads to a better sleep quality and therefore to more reliable results. A previous interview of untrained volunteers regarding ergonomics and user-friendliness of the monitor showed a positive result [17]. The SE classification can be used to create a simple traffic light system (e.g. green meaning “everything is fine”, and red meaning “see a doctor”), understandable without medical knowledge. It can therefore be assumed that the new sleep monitor can be used for simple sleep monitoring and preliminary screening at home. Additionally, previous research proved that the new sleep monitor is also able to diagnose one of the most common sleep disorders OSA [19], [20]. Using sleep staging, it is suggested that the monitor can now also be used to diagnose other sleep disorders, such as insomnia, or for a preliminary screening to decide whether a PSG is necessary or not. However, further studies need to be carried out to validate the ease of use and the reliability of the new monitoring system in an unattended setting. In summary, the presented method provides high performance for 2- and 3-stage sleep staging while still maintaining a simple setup and a high comfort for the patient.

## Notes

### Competing interests

The authors declare that they have no competing interests.

### Clinical Trial

Trial name: Validation of a new method for ambulant diagnosis of sleep related breathing disorders using body sound.

Trial-ID: DRKS00011195

URL: https://www.drks.de/drks_web/navigate.do?navigationId=trial.HTML&TRIAL_ID=DRKS00011195

## Figures and Tables

**Table 1:**
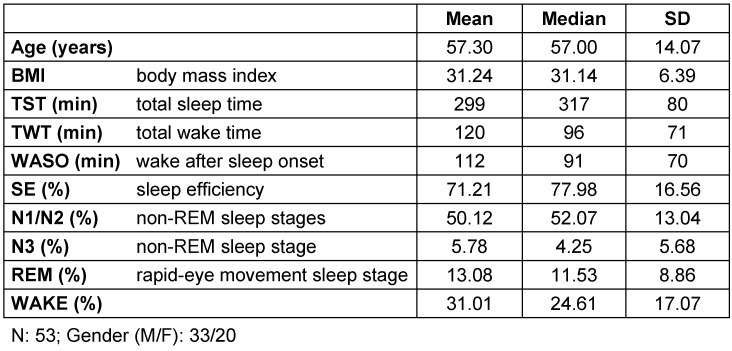
Anthropometric information of the subjects

**Table 2:**
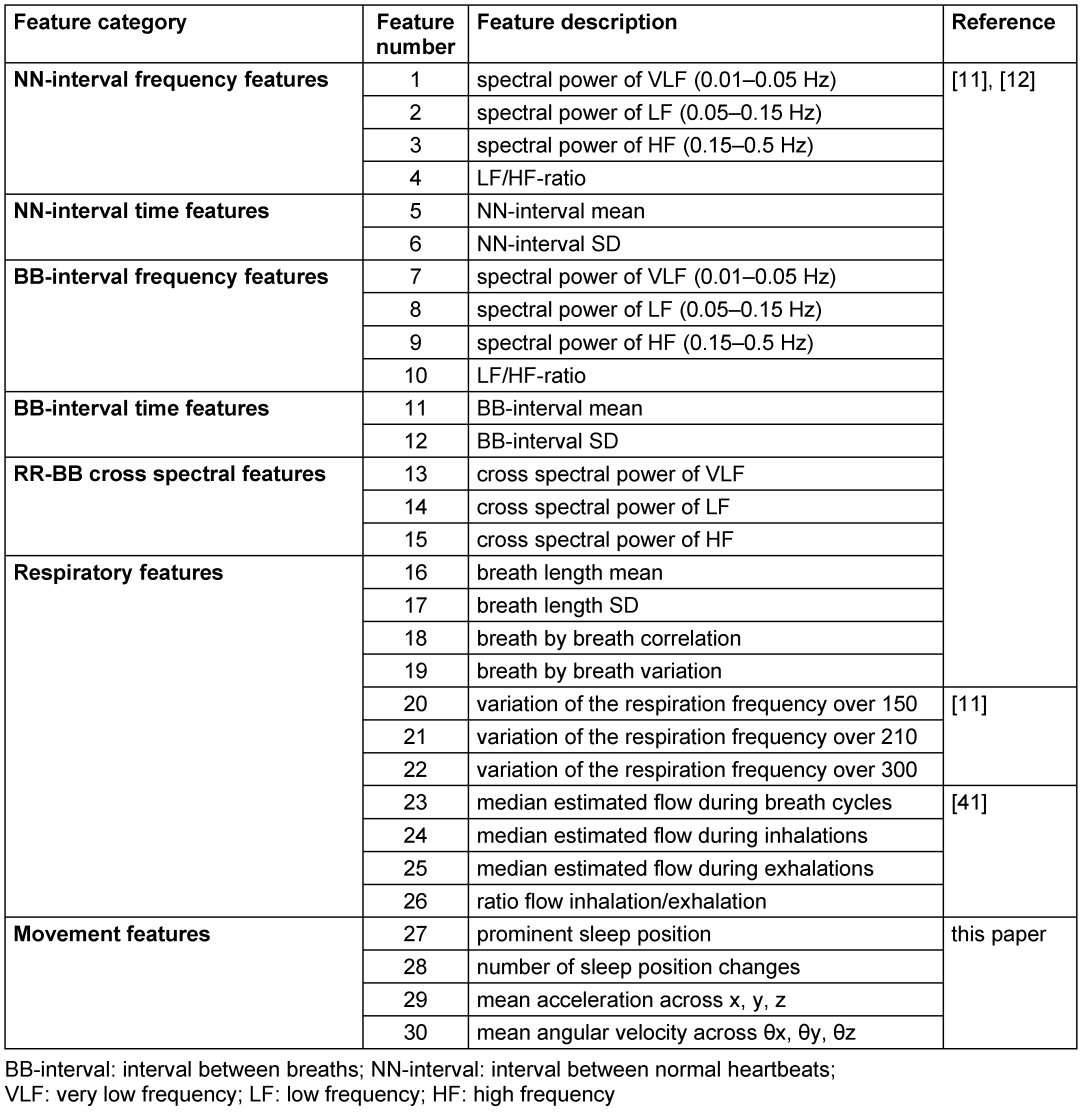
List of all features used for automated sleep staging

**Table 3:**
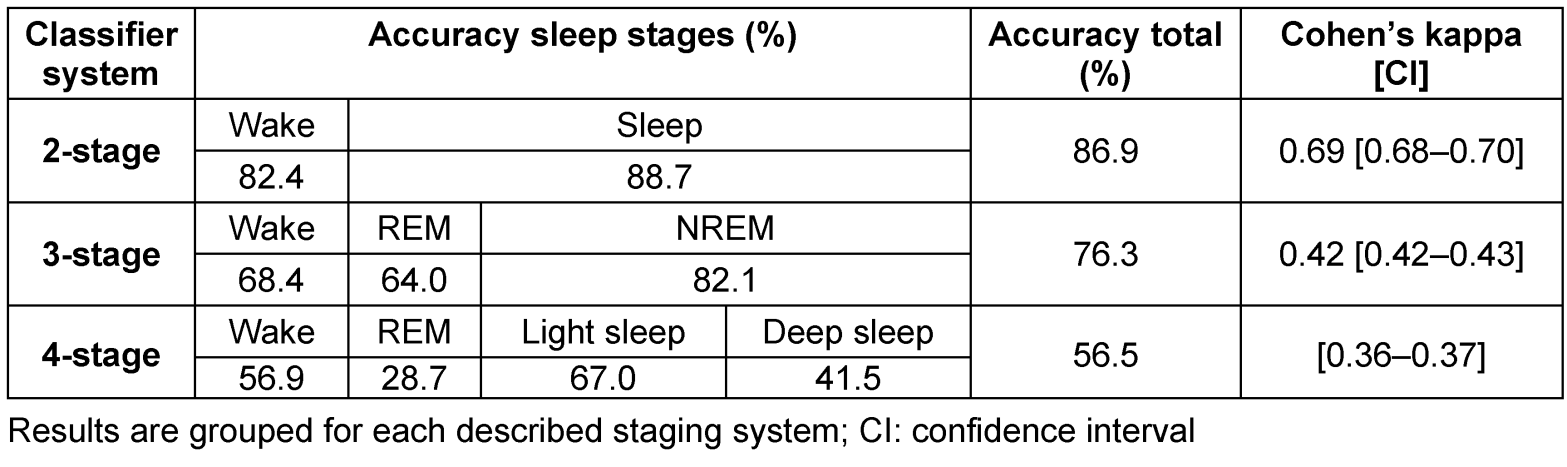
Evaluation of the 2-, 3-, 4-stage classifier performance for sleep staging

**Table 4:**
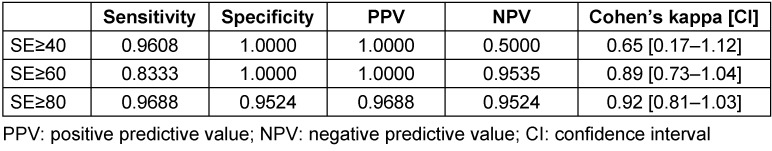
System performance based on the evaluation of the subject classification into different groups of sleep efficiency (SE) including all 53 subjects

**Table 5:**
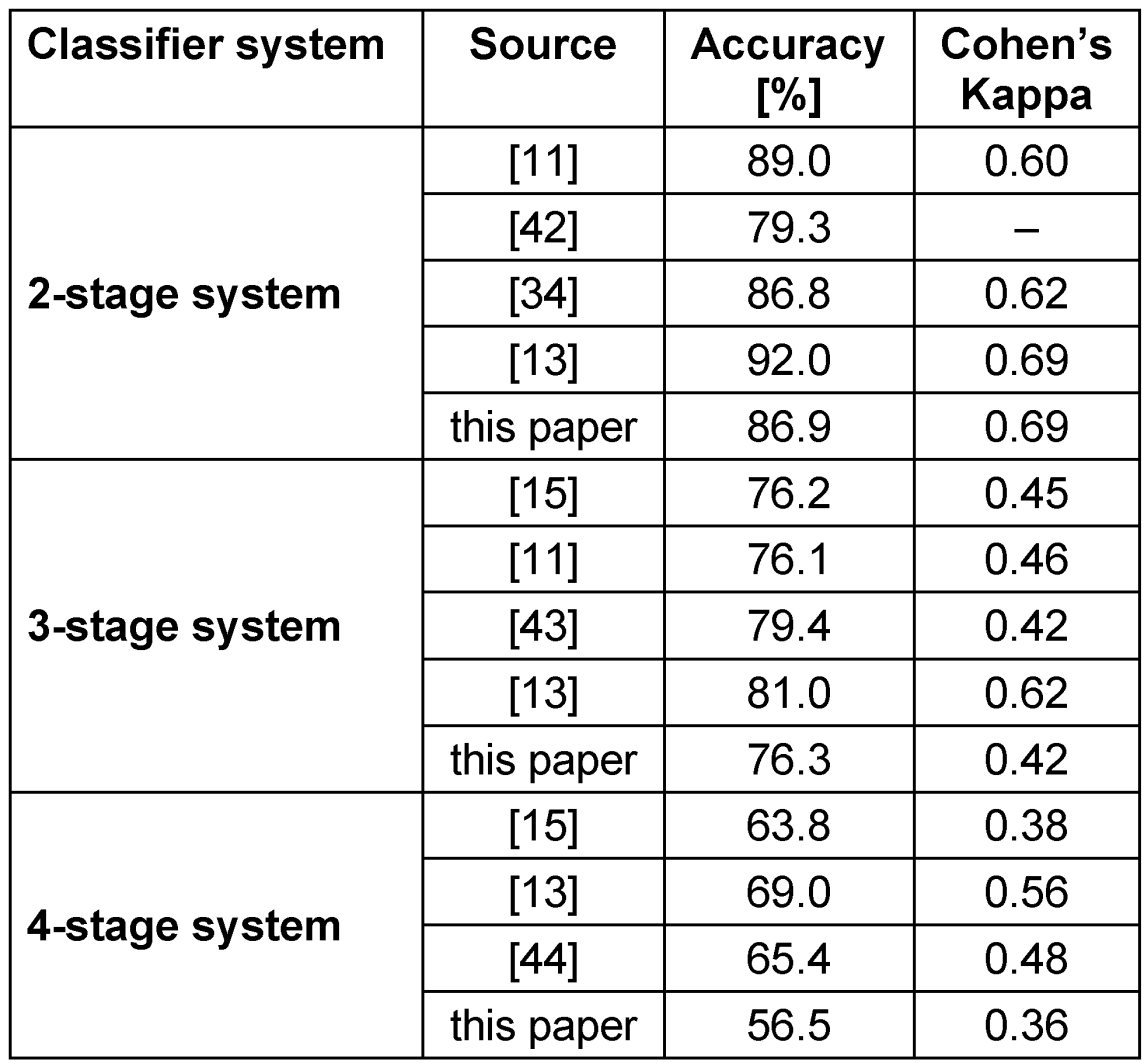
Overview of comparable sleep staging results found in literature

## References

[1] Cappuccio FP, Miller MA, Lockley SW (2010). Sleep, health, and society.

[2] Peppard PE, Young T, Barnet JH, Palta M, Hagen EW, Hla KM (2013). Increased prevalence of sleep-disordered breathing in adults. Am J Epidemiol.

[3] Rakel RE (2009). Clinical and societal consequences of obstructive sleep apnea and excessive daytime sleepiness. Postgrad Med.

[4] Penzel T, Blau A, Garcia C, Schöbel C, Sebert M, Fietze I (2012). Portable monitoring in sleep apnea. Curr Respir Care Rep.

[5] Corral-Peñafiel J, Pepin JL, Barbe F (2013). Ambulatory monitoring in the diagnosis and management of obstructive sleep apnoea syndrome. Eur Respir Rev.

[6] Ross SD, Sheinhait IA, Harrison KJ, Kvasz M, Connelly JE, Shea SA, Allen IE (2000). Systematic review and meta-analysis of the literature regarding the diagnosis of sleep apnea. Sleep.

[7] Collop NA, Anderson WM, Boehlecke B, Claman D, Goldberg R, Gottlieb DJ, Hudgel D, Sateia M, Schwab R (2007). Clinical guidelines for the use of unattended portable monitors in the diagnosis of obstructive sleep apnea in adult patients. Portable Monitoring Task Force of the American Academy of Sleep Medicine. J Clin Sleep Med.

[8] American Academy of Sleep Medicine (2014). International classification of sleep disorders.

[9] Rechtschaffen A, Kales A, University of California Los Angeles Brain Information Service, NINDB Neurological Information Network (US) (1968). A manual of standardized terminology, techniques and scoring system for sleep stages of human subjects.

[10] Silber MH, Ancoli-Israel S, Bonnet MH, Chokroverty S, Grigg-Damberger MM, Hirshkowitz M, Kapen S, Keenan SA, Kryger MH, Penzel T, Pressman MR, Iber C (2007). The visual scoring of sleep in adults. J Clin Sleep Med.

[11] Redmond SJ, de Chazal P, O’Brien C, Ryan S, McNicholas WT, Heneghan C (2007). Sleep staging using cardiorespiratory signals. Somnologie.

[12] Redmond SJ, Heneghan C (2006). Cardiorespiratory-based sleep staging in subjects with obstructive sleep apnea. IEEE Trans Biomed Eng.

[13] Willemen T, Van Deun D, Verhaert V, Vandekerckhove M, Exadaktylos V, Verbraecken J, Van Huffel S, Haex B, Sloten JV (2014). An evaluation of cardiorespiratory and movement features with respect to sleep-stage classification. IEEE J Biomed Health Inform.

[14] Isler JR, Thai T, Myers MM, Fifer WP (2016). An automated method for coding sleep states in human infants based on respiratory rate variability. Dev Psychobiol.

[15] Long X, Foussier J, Fonseca P, Haakma R, Aarts RM (2014). Analyzing respiratory effort amplitude for automated sleep stage classification. Biomedical Signal Processing and Control.

[16] Long X, Yang J, Weysen T, Haakma R, Foussier J, Fonseca P, Aarts RM (2014). Measuring dissimilarity between respiratory effort signals based on uniform scaling for sleep staging. Physiol Meas.

[17] Kalkbrenner C, Eichenlaub M, Brucher R (2015). Development of a new homecare sleep monitor using body sounds and motion tracking. Current Directions in Biomedical Engineering.

[18] Kalkbrenner C, Stark P, Kouemou G, Algorri ME, Brucher R (2014). Sleep monitoring using body sounds and motion tracking. Conf Proc IEEE Eng Med Biol Soc.

[19] Kalkbrenner C, Eichenlaub M, Rüdiger S, Kropf-Sanchen C, Brucher R, Rottbauer W (2017). Validation of a New System Using Tracheal Body Sound and Movement Data for Automated Apnea-Hypopnea Index Estimation. J Clin Sleep Med.

[20] Kalkbrenner C, Eichenlaub M, Rüdiger S, Kropf-Sanchen C, Rottbauer W, Brucher R (2018). Apnea and heart rate detection from tracheal body sounds for the diagnosis of sleep-related breathing disorders. Med Biol Eng Comput.

[21] American Academy of Sleep Medicine (2007). Das AASM-Manual zum Scoring von Schlaf und assoziierten Ereignissen: Regeln, Technologie und technische Spezifikationen.

[22] Roebuck A, Monasterio V, Gederi E, Osipov M, Behar J, Malhotra A, Penzel T, Clifford GD (2014). A review of signals used in sleep analysis. Physiol Meas.

[23] Penzel T, Kantelhardt JW, Lo CC, Voigt K, Vogelmeier C (2003). Dynamics of heart rate and sleep stages in normals and patients with sleep apnea. Neuropsychopharmacology.

[24] Vanoli E, Adamson PB, Ba-Lin, Pinna GD, Lazzara R, Orr WC (1995). Heart rate variability during specific sleep stages. A comparison of healthy subjects with patients after myocardial infarction. Circulation.

[25] Cajochen C, Pischke J, Aeschbach D, Borbély AA (1994). Heart rate dynamics during human sleep. Physiol Behav.

[26] Bonnet MH, Arand DL (1997). Heart rate variability: sleep stage, time of night, and arousal influences. Electroencephalogr Clin Neurophysiol.

[27] Douglas NJ, White DP, Pickett CK, Weil JV, Zwillich CW (1982). Respiration during sleep in normal man. Thorax.

[28] Snyder F, Hobson JA, Morrison DF, Goldfrank F (1964). Changes in respiration, heart rate, and systolic blood pressure in human sleep. J Appl Physiol.

[29] Khalighi S, Sousa T, Oliveira D, Pires G, Nunes U (2011). Efficient feature selection for sleep staging based on maximal overlap discrete wavelet transform and SVM. Conf Proc IEEE Eng Med Biol Soc.

[30] Boll S (1979). Suppression of acoustic noise in speech using spectral subtraction. IEEE Trans Acoust.

[31] Alshaer H, Fernie GR, Maki E, Bradley TD (2013). Validation of an automated algorithm for detecting apneas and hypopneas by acoustic analysis of breath sounds. Sleep Med.

[32] Alshaer H, Fernie GR, Sejdic E, Bradley TD (2009). Adaptive segmentation and normalization of breathing acoustic data of subjects with obstructive sleep apnea.

[33] Madgwick SO, Harrison AJ, Vaidyanathan A (2011). Estimation of IMU and MARG orientation using a gradient descent algorithm. IEEE Int Conf Rehabil Robot.

[34] Devot S, Dratwa R, Naujokat E (2010). Sleep/wake detection based on cardiorespiratory signals and actigraphy. Conf Proc IEEE Eng Med Biol Soc.

[35] Ebrahimi F, Setarehdan SK, Ayala-Moyeda J, Nazeran H (2013). Automatic sleep staging using empirical mode decomposition, discrete wavelet transform, time-domain, and nonlinear dynamics features of heart rate variability signals. Comput Methods Programs Biomed.

[36] Landis JR, Koch GG (1977). The Measurement of Observer Agreement for Categorical Data. Biometrics.

[37] Long X, Haakma R, Leufkens TR, Fonseca P, Aarts RM (2015). Effects of Between- and Within-Subject Variability on Autonomic Cardiorespiratory Activity during Sleep and Their Limitations on Sleep Staging: A Multilevel Analysis. Comput Intell Neurosci.

[38] Samy L, Huang MC, Liu JJ, Xu W, Sarrafzadeh M (2014). Unobtrusive Sleep Stage Identification Using a Pressure-Sensitive Bed Sheet. IEEE Sensors J.

[39] Kortelainen JM, Mendez MO, Bianchi AM, Matteucci M, Cerutti S (2010). Sleep staging based on signals acquired through bed sensor. IEEE Trans Inf Technol Biomed.

[40] Ruehland WR, O’Donoghue FJ, Pierce RJ, Thornton AT, Singh P, Copland JM, Stevens B, Rochford PD (2011). The 2007 AASM recommendations for EEG electrode placement in polysomnography: impact on sleep and cortical arousal scoring. Sleep.

[41] Long X, Fonseca P, Foussier J, Haakma R, Aarts RM (2014). Sleep and wake classification with actigraphy and respiratory effort using dynamic warping. IEEE J Biomed Health Inform.

[42] Mendez MO, Matteucci M, Castronovo V, Strambi LF, Cerutti S, Bianchi AM (2010). Sleep staging from Heart Rate Variability: Time-varying spectral features and Hidden Markov Models. IJBET.

[43] Mendez MO, Matteucci M, Cerutti S, Aletti F, Bianchi AM (2009). Sleep staging classification based on HRV: time-variant analysis. Conf Proc IEEE Eng Med Biol Soc.

[44] Hedner J, White DP, Malhotra A, Herscovici S, Pittman SD, Zou D, Grote L, Pillar G (2011). Sleep staging based on autonomic signals: a multi-center validation study. J Clin Sleep Med.

